# Exercise prescription for weight management in obese adults at risk for osteoarthritis: synthesis from a systematic review

**DOI:** 10.1186/s12891-019-3004-3

**Published:** 2019-12-20

**Authors:** Dylan R. Barrow, Lauren M. Abbate, Max R. Paquette, Jeffrey B. Driban, Heather K. Vincent, Connie Newman, Stephen P. Messier, Kirsten R. Ambrose, Sarah P. Shultz

**Affiliations:** 1 School of Sport, Exercise and Nutrition, Massey University, Wellington, New Zealand; 2 Geriatric Research, Education, and Clinical Center, Rocky Mountain Regional VA Medical Center, Aurora, CO USA; 3 Department of Emergency Medicine, University of Colorado School of Medicine, Aurora, CO USA; 4 School of Health Studies, University of Memphis, Memphis, TN USA; 5 Division of Rheumatology, Allergy & Immunology, Tufts Medical Center, Boston, MA USA; 6 UF Health Sports Performance Center, Department of Orthopedics and Rehabilitation, University of Florida, Gainesville, FL USA; 7 Department of Medicine, Division of Endocrinology, Diabetes, and Metabolism, New York University School of Medicine, New York, NY USA; 8 Department of Health and Exercise Science, Wake Forest University, Winston-Salem, NC USA; 9 Osteoarthritis Action Alliance, Thurston Arthritis Research Center, University of North Carolina, Chapel Hill, NC USA; 10 Department of Kinesiology, Seattle University, 901 12th Avenue, Seattle, WA 98122 USA

**Keywords:** Obesity, Musculoskeletal pain, Physical function, Physical activity

## Abstract

**Background:**

The aim of this systematic review was to identify principles of exercise interventions associated with improved physical function, weight management or musculoskeletal pain relief among young and middle-aged adults with obesity and propose an evidence-based exercise prescription that could assist in secondary prevention of osteoarthritis.

**Methods:**

A structured electronic review was conducted using MEDLINE, PubMed, and SPORTDiscus. The search string included 1) “obes*” AND “exercise” AND “interven*” AND “musculoskeletal pain OR knee pain OR hip pain”. Studies 1) were randomized controlled trials of humans, with a non-exercise control, 2) included participants aged 18–50 years, and 3) had outcomes that included physical function, musculoskeletal pain, and/or body composition. Studies were excluded if participants had peri-menopausal status, cancer, or obesity-related co-morbidities. A recommended exercise prescription was developed based on common principles used in the included exercise interventions with greatest change in function or pain.

**Results:**

Seven studies were included. Similarities in exercise intensity (40–80% VO_2max_), frequency (three times per week), duration (30–60 min), and exercise mode (treadmill, cross-trainer, stationary bike, aquatic exercise) were observed in exercise interventions that resulted in improved physical function and/or pain, compared to non-exercise control groups.

**Conclusion:**

Common principles in exercise prescription for improvements in weight management, physical function and pain relief among otherwise healthy people with obesity. Exercise prescription including moderate intensity exercise for 30–60 min, three times per week can be considered an effective treatment for weight management and obesity-related musculoskeletal symptoms. Exercise should be recommended to at-risk individuals as part of secondary prevention of osteoarthritis.

## Background

Over the past few decades the prevalence of obesity and osteoarthritis (OA) have increased [1, 2] and become a greater burden to society. Nearly one-third of all US adults with obesity also have some form of arthritis, with OA being the most common [3]. The increased prevalence of obesity and OA is problematic, as both conditions are common contributors to disability [1, 4, 5] with significant personal and societal impact [6]. Obesity contributes to the development of lower extremity OA [7] through the mechanical loading of excessive body weight and the pro-inflammatory impact of adipose tissue. Hence, preventing obesity or reducing the burden of obesity are important targets for OA prevention.

While primary prevention strategies focus on minimizing the risk factor (i.e. obesity prevention), secondary prevention strategies focus on populations that are already at risk (i.e. people with obesity) [8]. Secondary prevention strategies for individuals with obesity and are at risk for OA include interventions that focus on weight management, as well as improvements in neuromuscular deficits. Particularly, exercise is commonly utilized by people with obesity and/or OA, to directly address the goals of secondary prevention via benefits to body composition (including weight loss, fat loss, and muscle preservation) and the musculoskeletal system. For example, resistance exercise training improves functional capacity and joint range of motion and reduces pain [9, 10]. Aerobic training (e.g. walking, cycling) improves exercise tolerance, weight loss and musculoskeletal pain in populations with obesity and OA [11, 12]. General exercise prescription guidelines for weight loss have been published and include information on how hard (intensity), long (duration), and often (frequency) to exercise, as well as what activities to perform (mode) [13]. However, the evidence has not yet been systematically reviewed and summarized to identify common elements of exercise interventions that will most effectively address positive changes in body composition while simultaneously improving physical function and pain. A systematic review of these interventions and development of an exercise prescription based on studies that examined pain, disability, and improved body composition would provide recommendations for individuals with obesity who are at risk for OA.

Thus the purpose of this systematic review of randomized clinical trials is to identify prescriptive principles of exercise interventions in people with obesity and specifically address obesity-related physical dysfunction and musculoskeletal pain. These findings will assist in developing exercise programming to optimize musculoskeletal health outcomes and help reduce OA risks in people with obesity.

## Methods

### Identification of studies

**Fig. 1:**
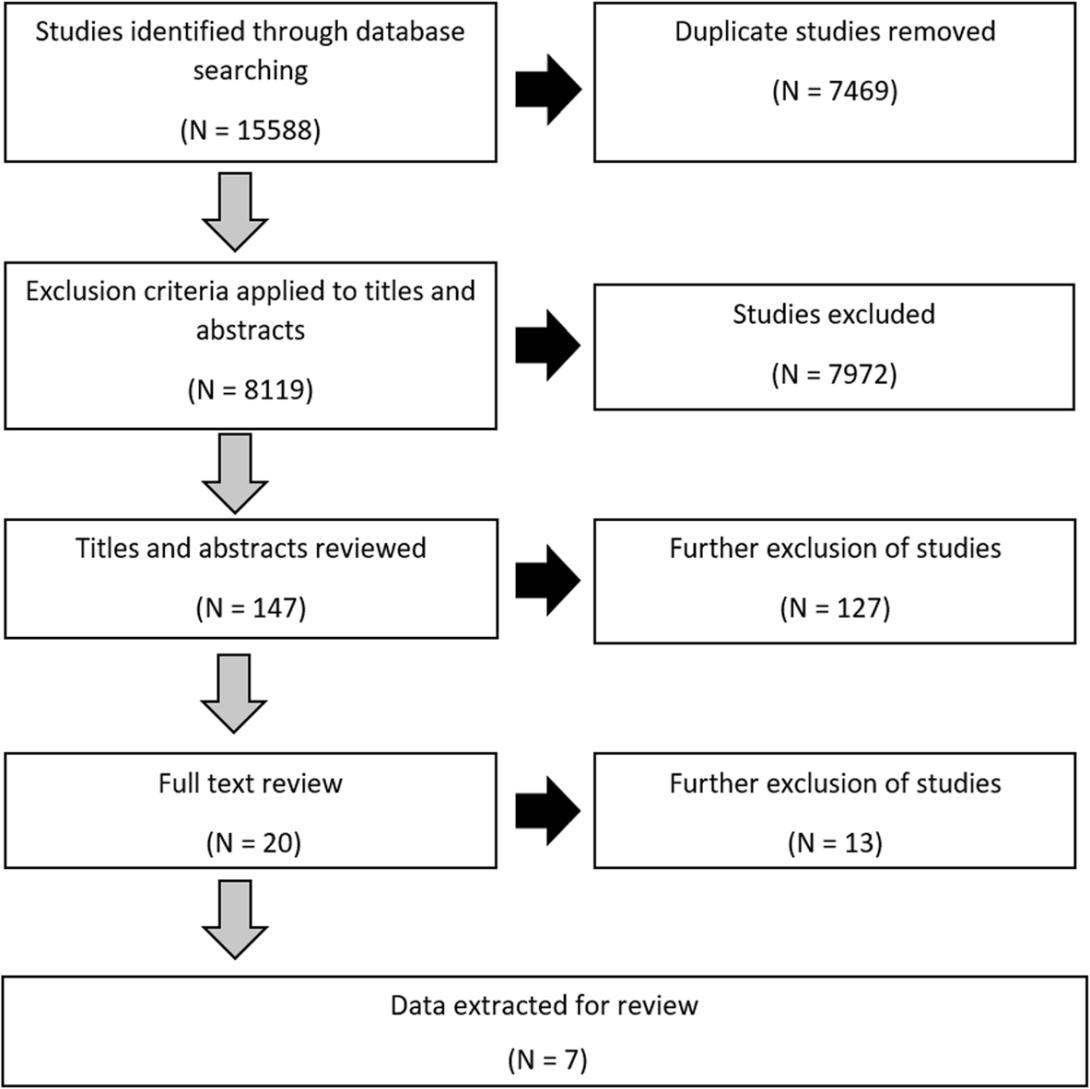
Evaluation of research studies during inclusion/ exclusion assessment

Figure 1 provides the protocol used to systematically assess the available literature. After consultation with a librarian, a structured electronic literature search was conducted between August 2017 and January 2018 using the following online databases: MEDLINE, PubMed, and SPORTDiscus. The search focused on populations with obesity and included the following terms: “obes*” AND “exercise” AND “interven*” AND “musculoskeletal pain OR knee pain OR hip pain”. Titles were then exported to a separate EndNote (Clarivate Analytics; Philadelphia, PA) file for processing. After duplicates were removed, the number of articles was reduced based on predefined inclusion and exclusion criteria.

### Inclusion and exclusion criteria

Only randomized controlled trials of humans published in the English language within peer-reviewed journals were considered for assessment. Conference proceedings, abstracts and theses were excluded. The first author (DB) assessed inclusion eligibility for all identified literature. First, titles and abstracts were screened for eligibility. Next, full text articles meeting initial screening eligibility were reviewed to ensure the articles were randomized controlled trials and met the inclusion/exclusion criteria outlined below. Where the exercise intervention was unclear [12], the corresponding author was contacted to seek further detail. Where a decision could not be reached by the investigating author, two other authors (SS and LA) were consulted to determine eligibility for inclusion. Inclusion/exclusion criteria based on study design, participants, intervention, and outcomes were established a priori:

1. Design: randomized controlled trials

### Participants

- To better focus on secondary prevention strategies, the study cohort included participants with a mean age between 18 and 50 years old.
- To avoid chronic illnesses that could confound the resulting benefits, participants had what is considered a healthy obese phenotype. Thus, articles with key words related to menopause status and obesity-related comorbidities (e.g., polycystic ovarian syndrome, cancer, stroke, diabetes, non-alcoholic [fatty] liver disease, cardiovascular conditions) were excluded.

2. Interventions: Studies must have had at least one non-exercise control group and one exercise-only group.
3. Outcomes: Outcomes focused on body composition addressed changes in mass, body mass index (BMI), and fat mass. All studies must have included either physical function or musculoskeletal pain as an outcome. Improvements in musculoskeletal pain and physical function were measured using objective measures of cardiorespiratory function and muscle properties, and validated subjective instruments such as the 36-Item Short Form Health Survey (SF-36) [14].

### Criteria for assessment of methodological quality

Authors (SM, JD, MP, CN, HV) assessed the methodological quality of each included study using a previously published and validated checklist by Downs and Black [15], which includes 26 items distributed between the following five subscales:

1. Reporting (9 items)
2. External validity (3 items)
3. Bias (7 items)
4. Confounding (6 items)
5. Power (1 item)

Briefly, the Reporting sub-section includes items to assess that the information provided in each paper was sufficient to allow the reader to assess the results from the article without bias. The External validity sub-section addresses whether findings from each article can be generalized to the population of the participants involved in the study. The Bias sub-section focuses on whether bias in the measurement of the outcome for each intervention exists in each article. The Confounding subsection examines whether there is bias in the selection of study participants. The Power sub-section determines if the negative findings from a study could be due to chance. Twenty four of the 26 items are scored as meeting [1] or not meeting (0) the item criteria. One item in the Power section required a scalar score between 0 and 5, based on the minimum number of participants in each group. Each article was scored by two independent reviewers who were blinded to each other’s scores to avoid any bias scoring; the maximum score for an article is 31. Downs and Black scores were classified as being excellent (31–29), good (28–23), fair (22–18), and poor (≤17) [16]. If there was a lack of consensus between the reviewers concerning the score classification (i.e. excellent, good, fair, poor) then a third reviewer acted as arbitrator to reach agreement.

## Results

Following the protocol presented in Fig. 1, the initial search resulted in 15,588 entries. We removed duplicates (7469) and an additional 7972 articles after an initial screening of titles or abstracts that did not meet the pre-determined eligibility criteria. Secondary review of abstracts excluded a further 127 studies. The full text of the remaining 20 articles were assessed against the inclusion and exclusion criteria; seven articles were accepted for review.

### Assessment of methodological quality

Only studies with randomized controlled trials research designs were included in this analysis. Table 1 presents the average overall score and methodological classification for each study. Study scores were lower than expected: five studies were classified as poor [14, 17, 18, 20, 21], and two studies classified as fair [19, 22]. The lowest score was 15 [17] and highest score was 20 [22]. Common deductions existed across all studies, due to the nature of exercise interventions. Studies were unable to blind researchers or participants, or conceal randomization allocation. Exercise interventions also traditionally recruit smaller sample sizes, and these studies rarely adjusted for confounding variables such as age, sex, or previous injury.

**Table 1:** Average Downs and Black scores for included studies

Reference	Downs & Black Score	Downs & Black Category
Arad et al. [17]	15	Poor
Blue et al. [18]	16	Poor
Chiu et al. [19]	18	Fair
Domene et al. [20]	17	Poor
Ross et al. [21]	16	Poor
Svensson et al. [14].	17	Poor
Utter et al. [22]	20	Fair

### Participant characteristics

There were 392 participants in the seven included studies (where reported: average age = 38 years, average BMI = 33.4 kg/m^2^; average body fat percent (BF%) = 36.4%). Of the 392 participants, 227 (100 Female, 59 Male, 69 Unknown) participants were allocated to the control or exercise intervention groups. Table 2 provides descriptive characteristics for each study.

**Table 2:** Descriptive characteristics of included studies

Study	Control	Exercise	Exercise Prescription
Arad, DiMenna, Thomas, et al. [17]	*N* = 11 (14) Age: 30 ± 7y BMI: 32.5 ± 3.6 BF%: 45.4 ± 3.7	*N* = 9 (14) Age: 29 ± 4y BMI: 32.1 ± 3.2 BF%: 45.4 ± 5.0	• Intervention = 14 weeks • Frequency = 3 x weekly • Exercise mode = cycling (HIT) • Intensity = 4 work intervals (30–60s) at 75–90% HRR. Recovery intervals between work intervals were 180–210 s at 50% HRR. Progressive overload until work rest ratio 60s:180s at intensity of 90% HRR for work intervals
Blue, Smith-Ryan, Trexler and Hirsch [18]	*N* = 9 Age: 37.0 ± 12.4 BMI: 33.6 ± 7.2 BF %: 33.4 ± 6.4	*N* = 16 Age: 31.4 ± 12.0y BMI: 31.8 ± 3.9 BF %: 32.6 ± 6.5	• Intervention = 3 weeks • Frequency = 3 x weekly • Exercise mode = cycling • Intensity = 10 reps of 1 min bouts at 90% peak power output; 1 min rest between work sets
Chiu, Ko, Wu, et al. [19]	N = 12 (14) Age: 20.8 ± 0.7 BMI: 30.38 ± 0.86 BF %: 33.3 ± 1.1	*N* = 12 (13) Age: 21.8 ± 0.7 BMI: 29.43 ± 0.56 BF %: 34.5 ± 1.8	• Intervention = 12 weeks • Session duration = 60 min • Frequency = 3 x weekly • Exercise mode = treadmill (aerobic) • Intensity = 40–80% HRR for weeks (increased gradually over 12 weeks)
Domene, Moir, Pummell, Knox and Easton [20]	*N* = 10 (11) Age: 35 ± 13 BMI: 27.6 ± 2.0 BF %: 31.7 ± 5.8	*N* = 10 (12) Age: 33 ± 11 BMI: 26.7 ± 1.7 BF %: 30.9 ± 5.5	• Intervention = 8 Weeks • Session duration = 1 h • Frequency = 12 x classes • Exercise mode = Aerobic • Intensity = Vigorous
Ross, Dagnone, Jones, et al. [21]	*N* = 8 (22) Age: 46.0 ± 10.9 BMI: 30.7 ± 1.6	*N* = 16 (27) Age: 45.0 ± 7.5 BMI: 32.3 ± 1.9	• Intervention = 12 weeks • Session duration = time to expend 700 kcal • Frequency = daily • Exercise mode = Treadmill (aerobic) • Intensity = no greater than 70% peak oxygen uptake
Svensson, Eek, Christiansen and Wisén [14]	*N* = 22 (31) Age: 47.4 ± 9.1 BMI: 44.7 ± 7.1	*N* = 49 (80) Age: 43.6 ± 8.3 BMI: 41.6 ± 5.2	• Intervention = 16 weeks • Session duration = 1 h • Frequency = 3 x weekly • Exercise mode = cycle, rowing machine, treadmill, cross-trainer (aerobic) + resistance training • Intensity (aerobic) = 6-min intervals at HR > 90%max. 30s pause between each bout • Intensity (resistance) = 2-min intervals at > 90% HRmax. 30s pause between each bout
Utter, Nieman, Shannonhouse, Butterworth and Nieman [22]	*N* = 22 Age: 43.7 ± 2.4 BMI: 32.8 ± 1.0	*N* = 21 Age: 44.6 ± 2.5 BMI: 32.3 ± 1.1	• Intervention = 12 weeks • Session duration = 45 min • Frequency = 5 x weekly • Exercise mode = walking (aerobic) • Intensity = 60–80% HRmax

Note. Sample size includes participants who completed the study and, where available, number of participants who were originally allocated (found in parentheses). *BMI* Body Mass Index, *HRR* Heart Rate Reserve, *BF%* Body Fat Percentage, *HIT* High Intensity Training, *HRmax* maximal heart rate

### Study interventions

Among the included studies, almost all control groups maintained existing and habitual activity or exercise without additional interference from the intervention. The lone exception was Utter et al. [22], which included a minimal intervention of stretching and light calisthenics. Exercise interventions for populations with obesity varied in duration, length, mode, and intensity (Table 2). Intervention durations ranged from three weeks [18] to 16 weeks [14]; an intervention duration of 12 weeks was most common [19, 21, 22]. Exercise session length varied from 20 to 90 min. The frequency of exercise varied from two times per week to daily; however, three times per week was the most common frequency [14, 17–19]. The mode of exercise varied among studies; however aerobic exercises were most common. Aerobic only exercise included weight bearing (treadmill, walking) [19, 21, 22], and partial weight bearing (cycling, aquatic exercise, elliptical and rowing ergometers) modalities [17, 18]. One study [14] combined aerobic and resistance training as part of their exercise intervention. Exercise intensity ranged from 40 to 90% of maximal heart rate for some interval-based training. Most studies required a vigorous intensity during aerobic exercise; resistance training was set at a similar intensity [14].

### Effect on outcome measures

Table 3 presents baseline and post-intervention data for control and intervention groups. Only outcomes that were significantly different before and after the exercise intervention were included.

**Table 3:** Outcomes with statistically significant post-intervention differences, as a result of completing the exercise intervention

Study	Body Composition (pre-measure vs post-measure; % change)	Physical Function (pre-measure vs post-measure; % change)
Arad, DiMenna, Thomas, et al. [17]	Visceral Adipose Tissue (L) • Control (1.3 ± 0.6 vs 1.2 ± 0.6; − 8%) • Exercise (1.3 ± 0.7 vs 1.1 ± 0.6; − 15%	Gas Exchange Threshold (L/min) • Control (0.87 ± 0.35 vs 0.91 ± 0.0.37; 5%) • Exercise (0.97 ± 0.23 vs 1.29 ± 0.34; 33%)
Blue, Smith-Ryan, Trexler and Hirsch [18]		Muscle cross-sectional area (cm^2^) • Control (22.18 ± 8.58 vs 21.84 ± 8.37; − 2%) • Exercise (21.93 ± 7.04 vs 25.10 ± 7.87; 14%)
Chiu, Ko, Wu, et al. [19]	Body Weight (kg) • Control (89.83 ± 4.38 vs 90.57 ± 4.40; 1%) • Exercise (84.38 ± 2.71 vs 77.66 ± 2.33; − 7%) Body Mass Index (kg/m^2^) • Control (30.38 ± 0.86 vs 30.64 ± 0.88; 1%) • Exercise (29.43 ± 0.56 vs 27.08 ± 0.39; − 8%) Body Fat (%) • Control (33.32 ± 1.07 vs 33.54 ± 1.20; 1%) • Exercise (34.50 ± 1.78 vs 30.53 ± 1.60; − 12%) Fat mass (kg) • Control (30.03 ± 1.99 vs 30.48 ± 2.07; 1%) • Exercise (29.04 ± 1.70 vs 23.60 ± 1.29; − 19%)	Cardiorespiratory Endurance Index • Control (55.30 ± 1.80 vs 54.78 ± 1.71; − 1%) • Exercise (56.82 ± 3.12 vs 64.24 ± 3.26; 13%)
Domene, Moir, Pummell, Knox and Easton [20]^a^	Body Fat (%) • Control (Δ = 0; 0%) • Exercise (Δ = -1.2; − 4%)	VO_2max_ (mL/kg/min) • Control (Δ = -0.7; − 3%) • Exercise (Δ = 3.1; 11%)
Ross, Dagnone, Jones, et al. [21]	Weight (kg) • Control (96.7 ± 9.0 vs 96.8; 0%) • Exercise (101.5 ± 7.7 vs 94.0; − 7%) Body Mass Index (kg/m^2^) • Control (30.7 ± 1.6 vs 30.7; 0%) • Exercise (32.3 ± 1.9 vs 29.9; − 7%) Total Fat (kg) • Control (30.5 ± 4.5 vs 29.9; − 2%) • Exercise (33.1 ± 5.5 vs 27.0; − 18%) Visceral Fat (kg) • Control (4.1 ± 1.7 vs 4.1; 0%) • Exercise (3.9 ± 1.0 vs 2.8; − 28%)	VO_2max_ (L/min) • Control (3.7 ± 0.8 vs 3.7; 0%) • Exercise (3.8 ± 0.8 vs 4.3; 12%)
Svensson, Eek, Christiansen and Wisén [14]		SF-36 Function (points) • Control (43.0 ± 8.2 vs 43.1 ± 10.5; 0%) • Exercise (48.6 ± 9.8 vs 50.5 ± 8.2; 4%)
Utter, Nieman, Shannonhouse, Butterworth and Nieman [22]		VO_2max_(ml/kg/min) • Control (22.2 ± 0.9 vs 23.2 ± 0.9; 5%) • Exercise (23.1 ± 0.7 vs 26.6 ± 0.9; 15%)

^a^Domene et al. provided change, rather than absolute values for pre and post anthropometric data

SF-36: 36-Item Short Form Survey. VO_2max_: Maximal Oxygen Uptake

#### Weight management

Three studies [19, 21, 22] reported statistically significant weight loss (weight loss range: 1–7.5 kg) and reductions in BMI (BMI reduction range: 0.3–2.34 kg/m^2^) after completing the exercise interventions. Five studies [17, 19–22] reported a statistically significant reduction in fat mass, as measured by dual x-ray absorptiometry [17], magnetic resonance imaging [21], and bioelectrical impedance analysis [19, 20]. The changes included reduced visceral adiposity (0.2 kg - 1.1 kg) [17, 21], total fat mass (5.44 kg - 7 kg) [19, 21, 22], and BF% (1–3.97%) [19, 20, 22]. Three studies that showed significant decreases in body/fat mass showed no significant change in skeletal or fat-free mass, indicating preservation of muscle mass [17, 19, 21]. High intensity exercise resulted in the greatest amount of weight loss [19] and reduced body/fat mass [17, 19], irrespective of exercise mode. Frequency and intervention duration were similar for both high intensity exercise interventions. Reductions in body/fat mass were also substantive during an aerobic intervention that required participants to exercise until they had expended 700 cal [21]. While some of the studies gradually progressed to vigorous intensity [19, 21, 22], all statistically significant body composition changes were seen in interventions with peak intensities ranging from 70 to 90% of maximal heart rate.

#### Pain

Although musculoskeletal pain was considered a variable of interest, only one study included a questionnaire addressing this variable [14]. Bodily pain was assessed as a sub-domain of SF-36; while differences were not significant, the high intensity exercise group (difference: 1.3 points) did improve their score where the control group’s average score worsened (difference: − 8.8 points).

#### Physical function

All included studies produced significant improvements in physical functioning compared to a control group. One study included both aerobic training and resistance training in their exercise interventions [14]. The high intensity exercise (> 90% maximal heart rate for aerobic and > 90% one-repetition maximum for resistance exercise) showed statistically significant improvement versus baseline testing for SF-36 Physical Function score [14].

The remaining six studies also indicated improvements in physical function but used interventions that included only aerobic exercise [17–22]. These interventions ranged in length from three weeks to 14 weeks and elicited other physiological changes that enhance functional capacity, including gas exchange threshold [17], muscle cross sectional area [18], and VO_2max_ [20–22]. For example, Arad et al. [17] reported improvements of 33% in gas exchange threshold compared to baseline after 14 weeks of exercise performed three times per week using high intensity interval training. Blue et al. [18] observed an increase in muscle cross sectional area of 14% following three weeks of exercise performed three times per week via the cycle ergometer at 90% peak power outlet. Improvements in VO_2max_ of 11, 13, and 15% respectively following interventions ranging from eight to 12 weeks. VO_2max_ increased irrespective of exercise mode (Zumba, treadmill walking, and walking on a track) [20–22]. Collective gains in gas exchange thresholds, muscle cross sectional area and VO_2max_ can increase muscle power and work rates, thus improving physical functional capacity.

## Discussion

The findings of this systematic review support the use of exercise for weight management and improvement of physical function in obese adults at risk for OA. We identified common exercise prescription parameters among successful exercise programs for individuals with obesity. Important parameters for exercise prescription include how hard (intensity), long (duration), and often (frequency) an individual should work out, as well as what type of activity to perform, to achieve optimal results for body composition (weight/fat loss and muscle preservation) and physical function. The following sections provide the basis for our recommendations.

### Recommended intensity: we recommend that individuals with obesity safely progress exercise up to vigorous intensities of approximately 70–80% of maximal heart rate to optimize weight management and improve physical function

The most effective exercise intensity for weight loss was implemented by Ross et al. [21], which induced an average weight loss of 7.5 kg (7% change) over 12 weeks when aerobic exercise (without diet) was completed at an intensity no greater than 70% VO_2peak_ until 700 kcal had been expended. Similar exercise intensities were used by Chiu et al. [19], resulting in similar weight loss (6.72 kg; 7% change) after aerobic exercise was completed three times each week at 40–80% of heart rate reserve for 12 weeks. Importantly, these changes in body mass occurred without any losses to muscle mass, thus preserving physical function while managing weight. Other studies [17, 20] found modest changes in fat mass, but continued to prescribe exercise between 60 and 90% of maximal heart rate. These findings are less surprising, given that cross-sectional research showed maximal fat oxidation occurs within the range of 50 and 70% VO_2max_ [23]. Because sedentary individuals do not often complete moderate intensity exercise, the increase to even the lower ranges of fat oxidation (i.e. 50% VO_2max_) [23] can result in modest improvements in body composition with less risk of inducing pain or discomfort. Exercise intensity can then be progressed up to 70% of VO_2max_ to achieve the greatest rate of fat oxidation [23]. The progression to higher exercise intensity will ultimately result in greater improvements to physical function and pain.

Recommendations for the optimal intensity for resistance training for populations with obesity is currently unclear as resistance training has not been shown to be effective in significantly reducing weight [13]. However, Svensson et al. [14] incorporated high intensity aerobic and high intensity resistance training into their intervention, which ultimately resulted in improved physical function scores. Since higher intensity resistance training has been associated with significant improvements in pain and function in OA populations [15, 24], we also recommend that resistance training at vigorous intensity be included as part of the secondary prevention strategy.

### Recommended frequency and duration: we recommend that individuals with obesity perform exercise two to three times per week with the goal of 30 to 60 min per session and increasing the frequency over time to maintain weight loss

Exercise frequency was similar between the different studies, with two to three times per week being the most common frequency prescribed [14, 17–19]. Using this frequency, these studies reported significant reductions in body weight, visceral adiposity, as well as improvements in cardiorespiratory function [17, 19], muscle cross-sectional area [18], and physical function [14]. Thus, the exercise frequency of two to three times per week appears to be effective in safely achieving exercise benefits for individuals with obesity. In exercise interventions lasting less than 16 weeks, a linear dose-response relationship occurs between exercise frequency and weight loss [25]. Higher exercise frequencies have also been associated with the prevention of weight gain over time [25]. Therefore, we recommend that individuals with obesity perform exercise two to three times per week with the goal of increasing this frequency over time to maintain weight loss.

Exercise sessions typically lasted between 30 and 60 min with the exception of Ross et al. [21], where session length was determined by the time to expend 700 kcal. The majority of reviewed studies [14, 19, 21, 22] prescribed exercise (60 min, three times per week) that fit within the recommendations for weight loss [13, 26]; however the consistent improvement in physical function suggests that these recommendations are also important for secondary prevention strategies.

### Recommended exercise mode: we recommend a variety of exercise modes can be used to maintain weight loss

Aerobic training was used in all reviewed studies, and did not differ greatly between interventions. Specifically, exercise conducted on treadmills, stationary bikes, rowing ergometers, or cross-trainers all yielded significant improvements in body composition and physical function. However, treadmill exercise demonstrated the greatest magnitude of reduction in weight and body fat [19, 21]. When resistance training was included [14], the exercises were multi-joint and machine-based. Based on the reviewed studies, a variety of aerobic modalities can be effective for secondary OA prevention. However, partial and non-weight bearing exercise such as cycling are still highly effective for improving body composition and physical function and can be included as an option when musculoskeletal pain prohibits/limits other modes.

### Limitations

Exercise interventions are inherently associated with limitations within a systematic review. Specifically, the inability to blind participants to the treatment will consistently result in lower scores when assessing methodological quality. Within this review, the number of studies included in the final data extraction was lower than what would have been anticipated. The low number of included studies, in combination with insufficient reporting of sex differences within some studies, prohibited any stratification by sex (a variable acknowledged for its ability to modify risk factors and intervention outcomes). The lower numbers could be a result of our exclusion of EMBASE from the search engines utilized. However, we believe that our exclusion criteria played a more significant role, primarily because we targeted variables (i.e. physical function, musculoskeletal pain) that are not commonly assessed in obesity research. Although overweight cohorts were not excluded from consideration, they were not actively pursued through search terms. Overweight individuals do carry an additional risk of developing OA and could have increased the number of papers included in the review. Due to the relatively low number of studies included, a meta-analysis was not feasible. Finally, recommendations may also be limited to otherwise healthy adults living with obesity, as our exclusion criteria removed many of the co-morbidities typically associated with both diseases. However, these recommendations offer guidance for clinicians and can provide a blueprint for future prevention trials.

## Conclusion

By identifying commonalities in the exercise prescription of individuals with a healthy obese phenotype, this review suggests an effective starting point in designing a secondary OA prevention strategy through exercise prescription. Only one study included resistance training, which found it to be most effective at high intensities, but can begin at a lower percentage of 1-repetition maximum and increase gradually, focusing on multi-joint movements and strengthening of the muscles surrounding the at-risk joints. Evidence suggests that aerobic exercise programs can also improve body composition (weight/fat loss with muscle preservation) and physical function among individuals with obesity. We propose that at the onset of exercise training, moderate intensity exercise should be prescribed with the intention to progress to levels of vigorous intensity (60–80% VO_2max_) as exercise tolerance increases. Exercise frequency and duration should be performed at least two to three times per week for at least 30–60 min for the greatest improvement to body composition and physical function. Exercise mode may include full (i.e. treadmill) or partial (i.e. cross-trainer, rowing ergometer, stationary bike) weight bearing exercises, depending on the pain symptoms, preferences, and physical function of the individual.

## Data Availability

Data sharing is not applicable to this article as no datasets were generated or analysed during the current study.

## Abbreviations

BF%: Body fat percentage
BMI: Body mass index
OA: Osteoarthritis
